# Buffalo Obstetrical Society

**Published:** 1884-03

**Authors:** 


### Buffalo Obstetrical Society.

We have in the city several Medical Societies devoted to general medicine. This society, devoted to Obstetrics, Gynæcology and Pediatrics, is the first organized for the study of a special class of medical topics. Its objects are similar to societies under the same title in other cities, and we bespeak for it like success. The following are its officers:

W. W. Potter, M. D., President.
R. L. Banta, M. D., Vice-President.
Geo. E. Fell, M. D., Sec. and Treas.

The meetings are held on the last Tuesday of each month. The first meeting was held at the residence of the President, Dr. Potter, 306 Franklin street, on Monday evening, Feb. 25th, in order to allow the members to attend the Medical Commencement which was to take place on its regular evening. Dr. Potter read a very interesting paper on Post Partum Hemorrhage, which called forth an interesting discussion. A bountiful collation fittingly supplemented the exercises of the evening. Dr. C. C. Fredericks is designated as the next essayist. The membership is limited to twenty members. We shall avail ourselves of the proceedings of this society for valuable material for the Journal. These special societies are successful in other cities, and we are confident the well-known ability of the projectors of this movement will secure a like success here.

